# Testing the Prognostic Accuracy of the Updated Pediatric Sepsis Biomarker Risk Model

**DOI:** 10.1371/journal.pone.0086242

**Published:** 2014-01-29

**Authors:** Hector R. Wong, Scott L. Weiss, John S. Giuliano, Mark S. Wainwright, Natalie Z. Cvijanovich, Neal J. Thomas, Geoffrey L. Allen, Nick Anas, Michael T. Bigham, Mark Hall, Robert J. Freishtat, Anita Sen, Keith Meyer, Paul A. Checchia, Thomas P. Shanley, Jeffrey Nowak, Michael Quasney, Arun Chopra, Julie C. Fitzgerald, Rainer Gedeit, Sharon Banschbach, Eileen Beckman, Patrick Lahni, Kimberly Hart, Christopher J. Lindsell

**Affiliations:** 1 Division of Critical Care Medicine, Cincinnati Children's Hospital Medical Center and Cincinnati Children's Research Foundation, Cincinnati, Ohio, United States of America; 2 Department of Pediatrics, University of Cincinnati College of Medicine, Cincinnati, Ohio, United States of America; 3 The Children's Hospital of Philadelphia, Philadelphia, Pennsylvania, United States of America; 4 Department of Pediatrics, Division of Critical Care Medicine, Yale University School of Medicine, New Haven, Connecticut, United States of America; 5 Ann & Robert H. Lurie Children's Hospital of Chicago, Chicago, Illinois, United States of America; 6 Children's Hospital and Research Center Oakland, Oakland, California, United States of America; 7 Penn State Hershey Children's Hospital, Hershey, Pennsylvania, United States of America; 8 Children's Mercy Hospital, Kansas City, Missouri, United States of America; 9 Children's Hospital of Orange County, Orange, California, United States of America; 10 Akron Children's Hospital, Akron, Ohio, United States of America; 11 Nationwide Children's Hospital, Columbus, Ohio, United States of America; 12 Children's National Medical Center, Washington, DC, United States of America; 13 Morgan Stanley Children's Hospital, Columbia University Medical Center, New York, New York, United States of America; 14 Miami Children's Hospital, Miami, Florida, United States of America; 15 Texas Children's Hospital, Houston, Texas, United States of America; 16 CS Mott Children's Hospital at the University of Michigan, Ann Arbor, Michigan, United States of America; 17 Children's Hospital and Clinics of Minnesota, Minneapolis, Minnesota, United States of America; 18 St. Christopher's Hospital for Children, Philadelphia, Pennsylvania, United States of America; 19 Children's Hospital of Wisconsin, Milwaukee, Wisconsin, United States of America; 20 Department of Emergency Medicine, University of Cincinnati College of Medicine, Cincinnati, Ohio, United States of America; University of Florida College of Medicine, United States of America

## Abstract

**Background:**

We previously derived and validated a risk model to estimate mortality probability in children with septic shock (PERSEVERE; PEdiatRic SEpsis biomarkEr Risk modEl). PERSEVERE uses five biomarkers and age to estimate mortality probability. After the initial derivation and validation of PERSEVERE, we combined the derivation and validation cohorts (n = 355) and updated PERSEVERE. An important step in the development of updated risk models is to test their accuracy using an independent test cohort.

**Objective:**

To test the prognostic accuracy of the updated version PERSEVERE in an independent test cohort.

**Methods:**

Study subjects were recruited from multiple pediatric intensive care units in the United States. Biomarkers were measured in 182 pediatric subjects with septic shock using serum samples obtained during the first 24 hours of presentation. The accuracy of PERSEVERE 28-day mortality risk estimate was tested using diagnostic test statistics, and the net reclassification improvement (NRI) was used to test whether PERSEVERE adds information to a physiology-based scoring system.

**Results:**

Mortality in the test cohort was 13.2%. Using a risk cut-off of 2.5%, the sensitivity of PERSEVERE for mortality was 83% (95% CI 62–95), specificity was 75% (68–82), positive predictive value was 34% (22–47), and negative predictive value was 97% (91–99). The area under the receiver operating characteristic curve was 0.81 (0.70–0.92). The false positive subjects had a greater degree of organ failure burden and longer intensive care unit length of stay, compared to the true negative subjects. When adding PERSEVERE to a physiology-based scoring system, the net reclassification improvement was 0.91 (0.47–1.35; p<0.001).

**Conclusions:**

The updated version of PERSEVERE estimates mortality probability reliably in a heterogeneous test cohort of children with septic shock and provides information over and above a physiology-based scoring system.

## Introduction

Heterogeneity is a major feature of pediatric septic shock, including widely variable mortality risk [1]. In the absence of tools to accurately assess mortality risk, clinicians have little objective information to benchmark septic shock outcomes, adjust for risk in analyses of clinical data, risk stratify patients for interventional clinical trials, and guide decisions on which patients need the most aggressive treatment, and which do not. We recently reported the derivation and validation of the pediatric sepsis biomarker risk model (PERSEVERE; PEdiatRic SEpsis biomarkEr Risk modEl) [2]. PERSEVERE was derived using a Classification and Regression Tree (CART) approach to predict 28-day mortality. The derivation selected five biomarkers and age, from among twelve biomarkers (serum proteins) and clinical variables potentially associated with outcome. Importantly, PERSEVERE was derived using data measured during the first 24 hours of presentation to the pediatric intensive care unit (PICU) with septic shock, which is an optimal time for risk stratification. In addition, participants were drawn from multiple centers in the United States [3]–[5].

Updating risk models using larger learning data sets can enhance generalizability and reliability. After the initial derivation and validation of PERSEVERE, we therefore combined the derivation and validation cohorts (n = 355) and updated PERSEVERE [2]. The purpose of the current study is to formally test the prognostic accuracy of the updated version of PERSEVERE using an independent test cohort, which is a critical next step after updating the model. The study is reported following the STARD (STAndards for the Reporting of Diagnostic accuracy studies) initiative [6].

## Methods

### Ethics statement and test cohort study subjects

The test cohort subjects were pooled from four sources, all of which used the same definition for septic shock [7]. The Institutional Review Boards (IRB) of each participating institution approved secondary use of biological specimens and clinical data: Cincinnati Children's Hospital Medical Center, The Children's Hospital of Philadelphia, Yale University School of Medicine, Ann & Robert H. Lurie Children's Hospital of Chicago, Children's Hospital and Research Center Oakland, Penn State Hershey Children's Hospital, Children's Mercy Hospital, Children's Hospital of Orange County, Akron Children's Hospital, Nationwide Children's Hospital, Children's National Medical Center, Morgan Stanley Children's Hospital, Columbia University Medical Center, Miami Children's Hospital, Texas Children's Hospital, CS Mott Children's Hospital at the University of Michigan, St. Christopher's Hospital for Children, and Children's Hospital of Wisconsin. Written consent was obtained from the parents or legal guardians of all subjects enrolled. None of the test cohort subjects were included in the original derivation or validation of PERSEVERE.

Eighty-seven subjects were included from an ongoing genomics study in pediatric septic shock being conducted at 17 participating institutions [8]–[17]. Briefly, children ≤10 years of age admitted to the PICU and meeting pediatric-specific criteria for septic shock were eligible for enrollment. After written informed consent from parents or legal guardians, serum samples were obtained within 24 hours of initial presentation to the PICU with septic shock. The current analysis included subjects enrolled between September 2011 and May 2013.

Sixty subjects were included from among those enrolled in an ongoing, quality improvement program at Cincinnati Children's Hospital Medical Center (CCHMC), Cincinnati, Ohio. The program uses PERSEVERE to benchmark septic shock outcomes for all patients admitted to the CCHMC PICU with septic shock. Enrollment procedures are identical to those described above, except that there is no age restriction and the CCHMC IRB has granted permission for waiver of informed consent. Serum samples are collected from residual blood samples in the clinical laboratory. Subjects from this source were enrolled between May 2012 and May 2013.

Nineteen subjects (age range: 8 days to 18 years) were participants in a prospective, observational study at Ann & Robert H. Lurie Children's Hospital of Chicago, Chicago, Illinois, evaluating nitric oxide metabolism and mitochondrial function in children with septic shock [18]. Of the 30 subjects with septic shock enrolled in that study, 19 had serum samples available for analysis. The current analysis included subjects enrolled between May 2009 and June 2010.

Sixteen subjects (age range: 2 to 20 years old) were participants in a prospective, observational study at Yale-New Haven Children's Hospital, New Haven, Connecticut, evaluating angiopoietin levels in children with septic shock [19]. Of the 17 subjects with septic shock enrolled in that study, 16 had serum samples available for analysis. The current analysis included subjects enrolled between September 2009 and December 2011.

### Study procedures

For all studies, annotated clinical and laboratory data were collected daily while the participant was in the PICU. Illness severity was calculated prospectively using the Pediatric Risk of Mortality (PRISM) score [20]. The number of organ failures during the initial 7 days of PICU admission was recorded using pediatric-specific criteria [7]. PICU free days were calculated by subtracting the actual PICU length of stay from a theoretical maximum PICU length of stay of 28 days. Patients with a PICU length of stay greater than 28 days and patients who died during the 28-day study period were classified as having zero PICU free days. All-cause mortality was tracked up to 28 days after meeting criteria for septic shock.

### Biomarkers

PERSEVERE includes C-C chemokine ligand 3 (CCL3), interleukin 8 (IL8), heat shock protein 70 kDa 1B (HSPA1B), granzyme B (GZMB), and matrix metallopeptidase 8 (MMP8). Serum concentrations of these biomarkers were measured using a multi-plex magnetic bead platform (MILLIPLEX™ MAP) designed for this project by the EMD Millipore Corporation (Billerica, MA). Biomarker concentrations were measured in a Luminex® 100/200 System (Luminex Corporation, Austin, TX), according the manufacturers' specifications. Assay performance data were previously published [2].

### Statistical Analysis

Initially, data are described using medians, interquartile ranges, frequencies, and percentages. Comparisons between groups used the Mann-Whitney U-test, Chi-square, or Fisher's Exact tests as appropriate. Descriptive statistics and comparisons used SigmaStat Software (Systat Software, Inc., San Jose, CA).

CART analysis was used to derive, validate, and update PERSEVERE (Salford Predictive Modeler v6.6, Salford Systems, San Diego, CA) [2], [21], [22]. Performance of the resulting decision tree in this new test cohort is reported using diagnostic test statistics with 95% confidence intervals computed using the score method as implemented by the VassarStats Website for Statistical Computation [23]. The net reclassification improvement (NRI) was used to estimate the incremental predictive ability of the biomarker-based model compared to using PRISM scores alone [24]. The NRI was computed using the R-package Hmisc.

## Results

### Characteristics of the derivation and validation cohorts

Table 1 describes the new test cohort (n = 182), and compares this to the previously published derivation cohort (n = 355). The test cohort had a higher median age and a greater proportion of subjects with no race reported. No other differences were observed. Within the test cohort, the only difference between survivors and non-survivors was the median PRISM score.

**Table 1:** Demographics and clinical characteristics of the derivation and test cohorts.

	Derivation Cohort			Test Cohort		
	All	Survivors	Non-survivors	All	Survivors	Non-survivors
N	355	314	41	182	158	24
Mortality (%)	11.5	–	–	13.2	–	–
Median days to death	–	–	3	–	–	4
(IQR)			(2–12)			(2–6)
Mean days to death ±SD	–	–	7.5±8.5	–	–	4.6±4.2
Median age years	2.4	2.5	1.9	5.5	5.6	5.0
(IQR)	(0.9–6.1)	(1.0–6.3)	(0.5–5.6)	(1.6–13.0)^2^	(1.7–13.1)	(0.6–9.0)
Median PRISM score	13	12	26	11	11	19
(IQR)^1^	(7–20)	(7–19)	(17–36)	(9–18)	(8–15)	(13–25)^3^
# of males (%)	207 (58)	183 (58)	24 (59)	94 (52)	81 (51)	13 (54)
# of females (%)	148 (42)	131 (42)	17 (41)	88 (48)	77 (49)	11 (46)
# for race (%)						
*Caucasian*	266 (75)	237 (75)	29 (71)	129 (71)	112 (71)	17 (71)
*African American*	54 (15)	48 (15)	6 (15)	20 (11)	18 (11)	2 (8)
*Other*	18 (5)	15 (5)	3 (7)	3 (2)	1 (1)	2 (8)
*Unreported*	17 (5)	14 (4)	3 (7)	30 (16)^2^	27 (17)	3 (13)
# with gram (+) bacteria (%)	97 (27)	85 (27)	12 (29)	54 (30)	46 (29)	8 (33)
# with gram (−) bacteria (%)	82 (23)	73 (23)	9 (22)	39 (21)	31 (20)	8 (33)
# with viral infection (%)	26 (7)	22 (7)	4 (10)	9 (5)	5 (3)	4 (17)
# with fungal infection (%)	9 (3)	8 (3)	1 (2)	7 (4)	7 (4)	0 (0)
# with no organism isolated (%)	144 (41)	129 (41)	15 (37)	80 (44)	75 (47)	5 (21)
# with any co-morbidity (%)	143 (40)	127 (40)	16 (39)	69 (38)	60 (38)	9 (38)
# with malignancy (%)	34 (10)	31 (10)	3 (7)	16 (9)	15 (9)	1 (4)
# with immune suppression (%)^4^	29 (8)	26 (8)	3 (7)	17 (9)	13 (8)	4 (17)

1 Nineteen subjects (18 survivors and 1 non-survivor) in the test cohort did not have available PRISM scores.

2 p<0.05 vs. test cohort.

3 p<0.05 vs. respective survivors.

4 Refers to patients with immune suppression not related to cancer (for example, those receiving immune suppressive medication for solid organ or bone marrow transplantation, or those with a primary immune deficiency).

### Testing the model

**Figure 1:**
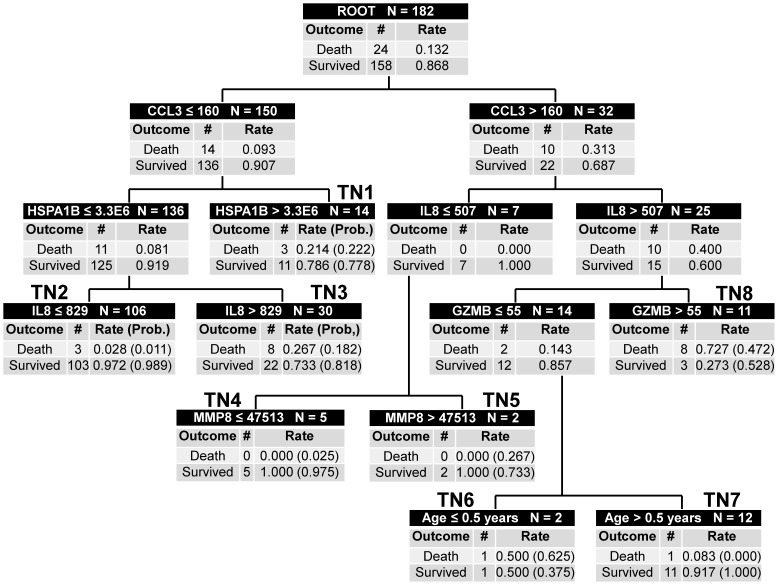
Classification of the test cohort subjects according to the updated version of PERSEVERE. The classification tree consists of 6 biomarker-based decision rules, 1 age-based decision rule, 14 daughter nodes, and 8 terminal nodes. The classification tree includes C-C chemokine ligand 3 (CCL3), heat shock protein 70 kDa 1B (HSPA1B), interleukin-8 (IL8), granzyme B (GZMB), and matrix metalloproteinase-8 (MMP8). Each node provides the total number of subjects in the node, the biomarker serum concentration- or age-based decision rule, and the number of survivors and non-survivors with the respective rates. For consistency, the serum concentrations of all stratification biomarkers are provided in pg/ml. The terminal nodes are numbered TN1 through TN8, and each terminal node provides the actual mortality and survival rates for the respective test cohort subjects, as well as the respective mortality probability of the updated decision tree, in parentheses. Terminal nodes 2, 4, and 7 are low risk terminal nodes (mortality probability 0.000 to 0.025), terminal nodes 1, 3, and 5 are intermediate risk terminal nodes (mortality probability 0.182 to 0.267), and terminal nodes 6 and 8 are high-risk terminal nodes (mortality probability 0.472 to 0.625). To calculate the diagnostic test characteristics, all subjects in the low risk terminal nodes (n = 123) were classified as predicted survivors, whereas all subjects in the intermediate and high risk terminal nodes (n = 59) were classified as predicted non-survivors.

The test cohort subjects were classified based on the decision rules of the updated model, without any modifications. Figure 1 shows the classification of the test cohort subjects according to the updated decision tree, which includes three low risk terminal nodes (TN2, TN4, and TN7; mortality probability 0.000 to 0.025), three intermediate risk terminal nodes (TN1, TN3, and TN5; mortality probability 0.182 to 0.267), and two high-risk terminal nodes (TN6 and TN8; mortality probability 0.472 to 0.625). There were 123 test cohort subjects classified as low risk and 59 subjects classified as either intermediate or high risk. Among the low risk subjects, four (3.3%) had died by 28 days. Among the intermediate and high-risk subjects 20 (33.9%) had died by 28 days. Table 2 shows the diagnostic test characteristics of the decision tree in the test cohort.

**Table 2:** Diagnostic test characteristics of the decision tree.

Number of Subjects	182
Overall Predicted Mortality	9.3% (7.2–11.3)
Number of True Positives	20
Number of True Negatives	119
Number of False Positives	39
Number of False Negatives	4
Sensitivity	83% (62–95)
Specificity	75% (68–82)
Positive Predictive Value	34% (22–47)
Negative Predictive Value	97% (91–99)
+Likelihood Ratio	(2.4–4.7)
−Likelihood Ratio	0.2 (0.1–0.5)
Area Under the Curve	0.811 (0.704–0.917)

When adding the information in PERSEVERE to the information in PRISM, the NRI was 0.906 (95% CI: 0.465–1.350; p<0.001). The NRI is a measure of how much the accuracy of predicted outcomes is improved when adding information [24]. The NRI ranges between −2 and +2. A score of −2 indicates that all true positives are reclassified as false negatives and all true negatives are reclassified as false positives, and no false classifications are reclassified as true classifications. Conversely, when the score is 2, adding the information correctly reclassifies every case. Our results demonstrate that the PERSEVERE provides significant additional classification value beyond the information included in PRISM.

### Secondary considerations

In our prior study, we noted that subjects classified as false positives (i.e. those predicted to die, but who actually survived) had greater illness severity than the true negative subjects (i.e. those predicted to survive, who did survive), as measured by PICU length of stay and organ failure burden [2]. We conducted a similar secondary analysis for the current test cohort. Table 3 shows that the false positive subjects in the test cohort had greater illness severity than the true negative subjects as measured by PICU length of stay, PICU free days, organ failure burden, and organ failure duration.

**Table 3:** Clinical course of the true negatives and the false positives.

	True Negatives	False Positives	p value
Number	119	39	
Median PICU length of stay, days (IQR)	4 (3–9)	7 (4–13)	0.005
Median PICU free days (IQR)	24 (19–25)	21 (15–24)	0.005
Median maximum number of organ failures (IQR)	1 (1–2)	3 (2–3)	<0.001
Number with ≥2 organ failures at PICU day 7 (%)	12 (10)	18 (46)	<0.001

## Discussion

Risk models require updating and ongoing prospective evaluation in order to enhance generalizability and acceptance. We have prospectively evaluated the prognostic accuracy of the updated version of PERSEVERE and found that it estimates mortality probability reliably in a heterogeneous test cohort. Among subjects predicted to be at intermediate or high-risk, the overall mortality rate was 33.9%, whereas subjects classified as low risk had an overall mortality rate of 3.3%. This dichotomous interpretation of PERSEVERE partitions a heterogeneous cohort of patients with septic shock into two groups having a ten-fold difference in mortality.

A more comprehensive view of PERSEVERE is to view each terminal node in the decision tree, and to assign individual patients with a mortality risk based on the probability of death in that terminal node. This allows for assigning a range of clinically relevant mortality probabilities and the ability to partition patients into low, intermediate, and high-risk groups. Moreover, the current validation study also demonstrates that PERSEVERE adds significant prognostic value to a physiology-based scoring system.

PERSEVERE generates reliable mortality risk prediction, but is imperfect; 21% of the test cohort subjects were false positives. This is to be expected if therapeutic interventions modify the outcomes of higher risk patients; the false positive subjects likely represent patients for whom therapeutic interventions prevented the predicted death. Support for this assertion is provided by our secondary analysis of the false positive and true negative patients. False positive subjects had a greater burden and duration of organ failure, and a greater PICU length of stay than true negative subjects, suggesting that PERSEVERE accurately identified higher acuity patients. Thus, even when the prediction is a false positive the information is likely clinically relevant.

The current test cohort was significantly older than the derivation cohort with almost one-third of the subjects being greater than 10 years of age. The issue of age is particularly important since the original derivation of PERSEVERE was based exclusively on children less than or equal to 10 years of age, and because developmental age strongly influences the host response during septic shock [13], [25]. As well as expanding the likely generalizability of PERSEVERE to subjects greater than 10 years, the test cohort was pooled from four different sources, each having its own unique potential for selection bias. This suggests that PERSEVERE has the potential for both broad applicability in pediatric septic shock, as well as having the potential to perform reliably in future prospective testing.

PERSEVERE has various potential clinical applications. First, it can be used as a benchmark to objectively evaluate septic shock outcomes. Poor outcomes in patients with a low PERSEVERE-based mortality risk, could suggest clinical underperformance and the need to review the clinical care process, while good outcomes in patients with a high PERSEVERE-based mortality risk could indicate better than expected clinical performance. We note that the actual mortality rate of the test cohort (13.3%) was higher than the overall mortality predicted by PERSEVERE (9.3%; 95% CI 7.2 to 11.3). This discrepancy reflects the four false negative classifications in terminal nodes 2 and 7. Three of these deaths were attributable to a single center, and in the quality peer review of these subjects, it was deemed that the deaths were unlikely to reflect a deficit in the care process. All three subjects had “do not resuscitate” orders in place and died after removal of advanced life support upon determination by the family and health care team that further support was futile. Two subjects had chronic multi-organ dysfunction associated with complications following bone marrow transplantation. The third subject had a lethal, progressive neurodegenerative disease. This illustrates how PERSEVERE can lead to a quality review of the care process, and the challenges inherent to assigning a mortality probability in patients with complex co-morbidities. Future calibrations of PERSEVERE may require the consideration of a co-morbidity variable, including immune function status. We note, however, that many test cohort subjects with significant co-morbidities (n = 45) or immune suppression (n = 12) were correctly classified by PERSEVERE.

Second, PERSEVERE could be used to conduct risk-stratified analyses of clinical data, as demonstrated in a recent study by our group [26]. We found the influence of positive fluid balance on pediatric septic shock outcomes to depend on risk as predicted by PERSEVERE. A positive fluid balance was associated with poor outcomes in the low mortality risk group, but not in the intermediate or high mortality risk groups. Third, PERSEVERE could be used to stratify patients for interventional clinical trials, and possibly to inform individual patient decision-making. These two latter applications will require the development of a rapid assay platform to generate biomarker data in a timely manner. No assay platform currently exists, but the technology to develop is readily available.

In conclusion, we have taken an important next step in the development of PERSEVERE. We have prospectively tested the prognostic accuracy of the updated version of PERSEVERE and found that it can be used to assign a reliable mortality probability in children with septic shock. This tool has various potential applications in the field of pediatric septic shock.

## References

[1] Hanna W, Wong HR (2013) Pediatric sepsis: challenges and adjunctive therapies. Crit Care Clin 29: 203–222. 23537672 10.1016/j.ccc.2012.11.003 PMC3612267

[2] Wong HR, Salisbury S, Xiao Q, Cvijanovich NZ, Hall M, et al (2012) The pediatric sepsis biomarker risk model. Crit Care 16: R174. 23025259 10.1186/cc11652 PMC3682273

[3] Kaplan JM, Wong HR (2011) Biomarker discovery and development in pediatric critical care medicine. Pediatr Crit Care Med 12: 165–173. 20473243 10.1097/PCC.0b013e3181e28876 PMC2924462

[4] Wong HR (2013) Genome-wide expression profiling in pediatric septic shock. Pediatr Res 73: 564–569. 23329198 10.1038/pr.2013.11 PMC3615026

[5] Standage SW, Wong HR (2011) Biomarkers for pediatric sepsis and septic shock. Expert Rev Anti Infect Ther 9: 71–79. 21171879 10.1586/eri.10.154 PMC3033193

[6] Bossuyt PM, Reitsma JB, Bruns DE, Gatsonis CA, Glasziou PP, et al (2003) Towards complete and accurate reporting of studies of diagnostic accuracy: The STARD Initiative. Ann Intern Med 138: 40–44. 12513043 10.7326/0003-4819-138-1-200301070-00010

[7] Goldstein B, Giroir B, Randolph A (2005) International pediatric sepsis consensus conference: definitions for sepsis and organ dysfunction in pediatrics. Pediatr Crit Care Med 6: 2–8. 15636651 10.1097/01.PCC.0000149131.72248.E6

[8] Cvijanovich N, Shanley TP, Lin R, Allen GL, Thomas NJ, et al (2008) Validating the genomic signature of pediatric septic shock. Physiol Genomics 34: 127–134. 18460642 10.1152/physiolgenomics.00025.2008 PMC2440641

[9] Shanley TP, Cvijanovich N, Lin R, Allen GL, Thomas NJ, et al (2007) Genome-level longitudinal expression of signaling pathways and gene networks in pediatric septic shock. Mol Med 13: 495–508. 17932561 10.2119/2007-00065.Shanley PMC2014731

[10] Wong HR, Cvijanovich N, Allen GL, Lin R, Anas N, et al (2009) Genomic expression profiling across the pediatric systemic inflammatory response syndrome, sepsis, and septic shock spectrum. Crit Care Med 37: 1558–1566. 19325468 10.1097/CCM.0b013e31819fcc08 PMC2747356

[11] Wong HR, Cvijanovich N, Lin R, Allen GL, Thomas NJ, et al (2009) Identification of pediatric septic shock subclasses based on genome-wide expression profiling. BMC Med 7: 34. 19624809 10.1186/1741-7015-7-34 PMC2720987

[12] Wong HR, Shanley TP, Sakthivel B, Cvijanovich N, Lin R, et al (2007) Genome level expression profiles in pediatric septic shock indicate a role for altered zinc homeostasis in poor outcome. Physiol Genomics 30: 146–155. 17374846 10.1152/physiolgenomics.00024.2007 PMC2770262

[13] Wynn JL, Cvijanovich NZ, Allen GL, Thomas NJ, Freishtat RJ, et al (2011) The influence of developmental age on the early transcriptomic response of children with septic shock. Mol Med 17: 1146–1156. 21738952 10.2119/molmed.2011.00169 PMC3321808

[14] Basu RK, Standage SW, Cvijanovich NZ, Allen GL, Thomas NJ, et al (2011) Identification of candidate serum biomarkers for severe septic shock-associated kidney injury via microarray. Crit Care 15: R273. 22098946 10.1186/cc10554 PMC3388679

[15] Wong HR, Cvijanovich NZ, Allen GL, Thomas NJ, Freishtat RJ, et al (2011) Validation of a gene expression-based subclassification strategy for pediatric septic shock. Crit Care Med 39: 2511–2517. 21705885 10.1097/CCM.0b013e3182257675 PMC3196776

[16] Wong HR, Freishtat RJ, Monaco M, Odoms K, Shanley TP (2010) Leukocyte subset-derived genomewide expression profiles in pediatric septic shock. Pediatr Crit Care Med 11: 349–355. 20009785 10.1097/PCC.0b013e3181c519b4 PMC2927840

[17] Wong HR, Cvijanovich N, Wheeler DS, Bigham MT, Monaco M, et al (2008) Interleukin-8 as a stratification tool for interventional trials involving pediatric septic shock. Am J Respir Crit Care Med 178: 276–282. 18511707 10.1164/rccm.200801-131OC PMC2542425

[18] Weiss SL, Haymond S, Ralay Ranaivo H, Wang D, De Jesus VR, et al (2012) Evaluation of asymmetric dimethylarginine, arginine, and carnitine metabolism in pediatric sepsis. Pediatr Crit Care Med 13: e210–218. 22460770 10.1097/PCC.0b013e318238b5cd PMC3392424

[19] Giuliano JS Jr, Tran K, Li FY, Northrup V, Tala JA, et al (2013) The temporal kinetics of circulating angiopoietin levels in children with sepsis. Pediatr Crit Care Med Oct 17 [Epub ahead of print]. 10.1097/PCC.0b013e3182a553bb PMC3947338 24141659

[20] Pollack MM, Patel KM, Ruttimann UE (1997) The Pediatric Risk of Mortality III–Acute Physiology Score (PRISM III-APS): a method of assessing physiologic instability for pediatric intensive care unit patients. J Pediatr 131: 575–581. 9386662 10.1016/s0022-3476(97)70065-9

[21] Che D, Liu Q, Rasheed K, Tao X (2011) Decision tree and ensemble learning algorithms with their applications in bioinformatics. Adv Exp Med Biol 696: 191–199. 21431559 10.1007/978-1-4419-7046-6_19

[22] Muller R, Mockel M (2008) Logistic regression and CART in the analysis of multimarker studies. Clin Chim Acta 394: 1–6. 18455512 10.1016/j.cca.2008.04.007

[23] Lowry R (2013) VassarStats Website for Statistical Computation. Available: http://faculty.vassar.edu/lowry/VassarStats.html. Accessed 2013 Dec 16.

[24] Steyerberg EW, Vickers AJ, Cook NR, Gerds T, Gonen M, et al (2010) Assessing the performance of prediction models: a framework for traditional and novel measures. Epidemiology 21: 128–138. 20010215 10.1097/EDE.0b013e3181c30fb2 PMC3575184

[25] Wynn J, Cornell TT, Wong HR, Shanley TP, Wheeler DS (2010) The host response to sepsis and developmental impact. Pediatrics 125: 1031–1041. 20421258 10.1542/peds.2009-3301 PMC2894560

[26] Abulebda A, Cvijanovich N, Thomas NJ, Allen GL, Anas N, et al (2013) Post-intensive care unit admission fluid balance and pediatric septic shock outcomes: A risk-stratified analysis. Crit Care Med Oct 18 [Epub ahead of print]. 10.1097/CCM.0b013e3182a64607 PMC3947064 24145842

